# Dental Hints Department

**Published:** 1906-02

**Authors:** B. H. Teague

**Affiliations:** Aiken, S. C.


### DENTAL HINTS DEPARTMENT.

Conducted by B. H. Teague, D. D. S., Aiken, S. C., to whom all contributions to this department should be sent.

Mummifying Paste.—Acetanelid and powdered alum, equal parts; glycerine, enough to make a stiff pasts, adding enough zinc oxide to make it smooth.—*Dr. C. L. Morey, Brief.*

I often tell my patients that there is one sovereign remedy for acute indigestion, and only one, viz., starvation.—*Holt, Clin. Med.*

"The Way of the Transgressor Is Hard."—With dental students operating on their teeth to pass a practical examination, the convicts in the Idaho penitentiary will renew their efforts to secure a pardon.—*Portland Telegram.*

Brewery employees are almost impervious to drugs and if sick seriously it generally fetches them.—*Phelps, Med. Summary.*

Neuralgia of face and head: Sp. gelsemium a drop every ten minutes.—Wherrell. Gelseminine, a granule as often, is better.—*Clin. Med.*

Odontalgia.—A pledget of cotton saturated with a solution of choral hydrate, 15 parts in 100 of glycerine, will stop pain almost instantly if introduced into the cavity of an aching tooth.—*Le Monde Dentaire.*

Canal Enlargement.—After years of study, practice, observation and experiment, I find root canal enlargement *unnecessary* when it is possible, and *impossible* when it seems necessary.—*W. C. Gowan, Dominion Dental Journal.*

Saliva-ejector Tubes.—I have had decided success in keeping my glass tubes for saliva ejection clean by permitting them to stand in a weak solution of sulphuric acid.—*W. G. Ebersole, The Dentist's Magazine.*

Myalgia.—When grandpa shows how strong he was 20 years ago and is laid up with "lumbago," give the ammonium chlor.—*Clin. Med.*

In operating on alcoholics remember low vitality of tissues; even moderate applications of heat may cause sloughing.—*I. J. S., Clin. Med.*

Before inserting a hypo needle, apply a drop of chloroform to the skin; it is antiseptic and anesthetic.—*Frank Pollard, Clin. Med.*

Porcelain Inlays; The Matrix.—The metal should never be annealed and the matrix introduced into the cavity after the final burnishing, as it is too easy a matter to distort the matrix in so doing.—*Craig M. Work, Dental Summary.*

Porcelain Crown.—Diatoric teeth make good bicuspid crowns, especially for lowers. Bake a pin into the tooth and it can then be adjusted with or without a band and finished in the usual way.—*Oliver Martin, in Review.*

The Rubber Dam.—A large field for operations should be provided for in the adjustment of the dam. Thoroughly desensitise the gum tissue and the rest is easy, both for the patient and operator.—*W. J. Jackman, The Dentist's Magazine.*

Vulcanizer Explodes.—The explosion of a vulcanizer in the office of Dr. W. R. Clark, at Youngstown, Ohio, almost resulted in his death. The top blew up, striking the ceiling above and throwing James A. Campbell, president of the Youngstown Sheet and Tube company, from the chair, so great was the force of the impact.—*Amer. D. Jour.*

To Stop Puncture in a Rubber Dam.—For punctures of the dam or to stop leakage around the lower anterior teeth, dry the dam with cotton, melt a little basel plate wax on a spatula and carry to points of leakage. This result will gratify you. —*Arthur C. Preston, Dental Quarterly.*

Preparing Sensitive Cavities.—The first application of the burr can be made absolutely painless in the most highly sensitive cavity by simply taking ethyl chlorid on the burr point and bringing it quickly into contact with the tooth.—*J. M. Gale, British Dental Journal.*

THE LOGAN CROWN.—I believe that for the majority of cases the Logan is the best form of porcelain crown, and that by burnishing the plate to a perfect joint to the end of the root, and filling in porcelain between, we have one of the very best crowns that can be made.—*C. L. Hungerford, Western Dental Journal.*

SEALING THE DENTAL TUBULI.—The right quantity of cement, mixed to a proper consistency, placed beneath gold foil in the cavity, and subjected to a pressure sufficient to weld the gold, will so perfectly seal the dental tubuli that we may entirely prevent the black rim of discoloration so prvalent between cohesive gold and the tooth substance, when the cavity is unlined.—*F. L. Trickey, Dental Review.*

EXPANSION OF RUBBER.—Black rubber expands the most by heat; pink the least. Pure rubber is the most apt to become spongy if thick pieces are overheated in vulcanizing. If stale from long keeping blow-holes or bubbles will be developed under the surface, even at low heat. Always buy fresh stock. —*Geo. B. Sword.*

DIED AFTER TAKING GAS.—George A. McDonough, a lithographer, forty-four years old, a resident in New York city, went to a dentist, Waldo Mork, to have an abscess treated. His family physician informed Dr. Mork that McDonough could safely take gas during the operation. When the work was done Dr. Mork could not arouse McDonough, who died half an hour later. Coroner Berry gave a certificate of death from heart disease.—*Bronx Sentinel.*

TO CEMENT ARSENIC IN CAVITY WITHOUT PRESSURE.—Mix the cement rather thin and place a small drop on a small bit of paper and carry the paper to the cavity with the pliers. Press to place with a burnisher. The paper facilitates adjustment to place and prevents cement adhering to instrument.—*Claude B. Warner, Dental Quarterly.*

A GOOD OBTUNDANT.—I find that a saturated solution of potassium carbonate in glycerine, as given in the September number of *The Dental Summary,* very effective for sensitive dentine. I apply to cavity on cotton and let remain from ten to fifteen minutes. It can be used freely in the open mouth, as it is non-poisonous.—*Dr. L. W. Jordan, Summary.*

Sealing Arsenic In Buccal Cavities.—Seal arsenious acid in shallow buccal cavities with wax, melting the surface around the margins with a hot, small spatula. A good preparation of wax for this purpose is made by using beeswax one part and resin sixteen parts.—*Oliver Martin, Review.*

Not In the Army, After All.—A Methodist negro exhorter shouted: "Come up en jine de army ob de Lohd."

"I'se done jined," replied one of the congregation.

"Whar'd yoh jine?" asked the exhorter.

"In de Baptis' Chu'ch."

"Why, chile," said the exhorter, "yoh ain't in the army; yoh's in de navy."—*Med. Clin. Jour.*

Porcelain Inlays Adhesion.—It has been demonstrated time and again that cement will not adhere to a glazed surface as well as to an etched surface. I have found from experience that in all cases where an inlay has dropped out of a cavity you will find that the cement adhered to the surface of the tooth, and not to the inlay itself. I do not attempt to groove my inlays any more on account of the danger of serious damage, and I find it is not necessary.—*J. E. Nyman, Dental Summary.*

Tooth Crowning: The Post.—Having carefully enlarged and shaped the root canal, and having the length of the canal marked on the hand instrument with which it has been prepared, an iridio-platinum post should be filed round and tapering to correspond in length and shape to accurately fit the canal. This should be done by the operator at the chair, and not in the laboratory, so that when the pin is slightly roughened, and the crown set to place, there will be no need of excess quantity of cement, an infinitesimally small amount only being needed to securely retain the crown in position.—*Burton Lee Thorpe, Le Laboratoire.*

Silver Nitrate.—Bryan of Basel has inaugurated a system of dental prophylaxis, in which he strongly advocates the application of a 40 per cent. nitrate of silver solution upon tooth surfaces slightly affected by the earlier stages of caries. With intervals of three months this process is to be repeated. He is very enthusiastic about his results, believing that the stimulating effect of the application causes the pulp to retract by depositing secondary dentin in the crown chamber.—*Hermann Prinz, Dental Digest.*

THE PORCELAIN CROWN.—I would advise the use of the 25 per cent. platinum solder when attaching the floor to the band and the dowel to the floor, especially if using a high-fusing body. If the dowel can be raised up a little above the floor it is stronger as regards the possibility of the crown tipping on the dowel. If the end of the dowel is flattened out on an anvil, spreading it to a dovetail, and given a little bend backward so as to get it back with the lingual cusp, it will greatly strengthen the crown.—*F. H. Berry, Dental Review.*

THE SENSE OF TOUCH.—In certain operations the highest development of the sense of touch is not only important but *vital* as well. For instance, in the treatment of pyorrhœa alveolaris, the removal of minute, deep-seated deposits which cannot be seen must precede the cure. The sense of touch is here as important as the sense of sight to the porcelain worker. In this treatment the other senses are secondary to the sense of touch, which is absolutely mental in its highest form.—*H. C. Spencer, Dental Cosmos.*

DENTAL EXHIBIT.—On March 27, 28, 29, 30, 1906, there will be held at the Chicago Auditorium the largest exhibit of dental goods and appliances ever attempted. There will be no lectures or papers, but experts in every line will give continuous demonstrations. There will be no admission fees or dues of any sort. A cordial invitation is extended to every dentist to attend.—*Amer. D. Jour.*

PROXIMAL CAVITY PREPARATION.—I am a great advocate of the occlusal step in proximal cavities. From the standpoint of convenience I find it an excellent thing, especially in the distal cavities of the molars; by extending a right angle step we can see into the cavity and be sure of a strong wall. It is difficult to get the anchorage in the buccal and lingual walls sufficiently strong and safe without some danger of tipping.—*Don M. Gallie, Review.*

ANTISEPTIC LIQUID SOAP.—White soap 1 kilogram, glycerin 250 grams, salol 60 grams, olive oil 250 grams, black soap 200 grams, water 3 liters. Boil for three hours and cool for one day, then add water 3 liters, decant, cool, and then add alcohol of 90° 250 grams. Put aside for two or three days and filter. These proportions are for five liters of liquid soap. I have found the plan which I now recommend very economical, as only a few drops of the liquid soap and antiseptic solution are required to thoroughly cleanse and sterilize the hands.—*Dr. Wormer, Cosmos.*

Treating Putrescent Canals.—Pulps having died from natural causes and becoming putrescent, and at times through some unknown cause, may develop blind abscesses. In treating, when there is apparent inflammation, make opening to give free access, then inject dioxogen freely and use either canal drill or broach to remove diseased pulp, continuing to use wash freely until you feel you have canal thoroughly cleansed. In some cases I use sulphuric acid diluted; apply with aluminum probe, then thoroughly disinfect and dry; fill with oxpara as formerly described and you can feel safe in filling tooth at same setting and expect no further trouble.

Should the case be one where the tooth has attained a state of abscess, then open and cleanse thoroughly as in former case and use acid; fill with oxpara, as formerly stated, and put in temporary filling, and paint gums with dental tinct. of aconite and iodine. If abscess develops, freely lance and use peroxide and you need expect no further trouble.—*Dr. C. G. Hughes, Summary.*

Meat Eating in the United States.—The American likes to talk of "the beef-eating Englishman," and his consequent choleric temperament, in blissful ignorance of the fact that in the consumption of meat the United States heads all nations. The enormous quantity of 11,000,000,000 pounds of meat—147 pounds to each person—is used here yearly. Of this amount, 5,000,000,000 pounds are beef, 4,000,000,000 pork, and 800,000,000 mutton. Great Britain stands next, with nearly one-third less—100 pounds per inhabitant. Norway uses 80 pounds; France, 77; Spain, 70; Germany, 64; Switzerland, 62; Belgium, 61; Austro-Hungary, 60; Russia, Portugal, and the Netherlands, 50 pounds each; Italy, 24 pounds.—*Good Health.*

Preparation to Stop Pain in a Tooth.—The following preparation has been found effective in relieving pain in a tooth where other remedies, usually employed, have produced no results.

Carbolic acid .................................dr. 1

Oil Sassafras .................................dr 2

Eugenol .................................dr. 2

4 per cent. solution Cocaine .................dr. 1

In having the preparation made up, be sure that eugenol is used and not oil of cloves.—*Dr. E. G. McAlrey, Review.*

